# Effect of dexmedetomidine on intraoperative Surgical Pleth Index in patients undergoing video-assisted thoracoscopic lung lobectomy

**DOI:** 10.1186/s13019-020-01346-1

**Published:** 2020-10-02

**Authors:** Yu-Lan Wang, Xiao-Qi Kong, Fu-Hai Ji

**Affiliations:** Department of Anesthesia Surgery, The First Affiliated Hospital of Soochow University, 188 Shizi Street, Suzhou, 215006 Jiangsu China

**Keywords:** Surgical Pleth index, Dexmedetomidine, Number rating scale, Thoracoscopic lung lobectomy

## Abstract

**Background:**

The Surgical Pleth Index (SPI) is a monitoring method that reflects painful stimuli during general anesthesia, and dexmedetomidine is an analgesic adjuvant with an opioid-sparing effect. But up to now, it is still unclear whether dexmedetomidine has any influence on SPI. To investigate whether dexmedetomidine has an effect on SPI during video-assisted thoracoscopic surgery.

**Methods:**

We enrolled 94 patients who underwent video-assisted thoracoscopic lung lobectomy. Patients were randomly assigned to a dexmedetomidine group (dexmedetomidine: 0.8 μg/kg administered for 10 min before anesthesia) or normal saline group (equal volume of normal saline). SPI and vital signs were recorded. The number rating scale (NRS) pain score was also evaluated.

**Results:**

SPI values were significantly lower in the dexmedetomidine group than in the normal saline group at intubation and at discharge from the postanesthesia care unit. Compared with the normal saline group, mean arterial pressure and heart rate were both significantly lower in the dexmedetomidine group at intubation. Heart rate was lower at skin incision in the dexmedetomidine group. The NRS score in the normal saline group was noticeably higher vs. the dexmedetomidine group at discharge from the postanesthesia care unit.

**Conclusions:**

Dexmedetomidine decreased intraoperative SPI and NRS scores. Our results showed that dexmedetomidine attenuated noxious stimuli.

**Trial registration:**

Chinese Clinical Trial Registry (ChiCTR): ChiCTR-OOC-16009450, Registered 16 October, 2016.

## Background

Video-assisted thoracoscopic surgery (VATS) is being performed more frequently. However, although thoracoscopic lung lobectomy is less traumatic than open thoracotomy, patients still experience significant pain [1, 2]. The Surgical Pleth Index (SPI), formerly, the “surgical stress index”, is a monitoring method for patients’ responses to surgical stimulation without injury. SPI can be used to monitor patients’ hemodynamic responses during general anesthesia because SPI reflects the increased sympathetic activity of the patient in response to painful (nociceptive) stimuli. The SPI value is obtained from photoplethysmographic amplitude (PPGA) and heart rate (HR) data from pulse oximetry measurements [3, 4]. Studies demonstrate that SPI can detect the balance between nociceptor activation and analgesia better than other parameters, including HR and blood pressure (BP) [5, 6]. Therefore, SPI can estimate intraoperative nociception. SPI ranges from 0 to 100, and higher values indicate stronger stimuli during surgery [7].

Dexmedetomidine is a short-acting a_2_-adrenoceptor agonist that provides sedation and analgesia [8, 9]. Studies show that dexmedetomidine attenuates surgical stress responses in patients undergoing surgery [10, 11], and, as an adjunctive analgesic, can be safely and effectively used during surgery. Currently, because of its characteristics, some anesthesiologists in clinical practice use dexmedetomidine as an auxiliary drug during anesthesia; however, other anesthesiologists do not use this drug because of a fear of significantly decreasing HR, and they choose other sedative drugs, such as midazolam.

Although thoracoscopic surgery is relatively less invasive, patients still experience moderate-intensity pain. SPI reflects intraoperative stress, and dexmedetomidine can weaken the surgical stress response. But up to now, it is still unclear whether dexmedetomidine has any influence on SPI. The aim of our study is to determine whether dexmedetomidine can reduce the SPI value during surgery, by investigating the effect of dexmedetomidine on SPI in patients undergoing VATS lung lobectomy.

## Methods

### Study population

This was a randomized, prospective clinical study. This study was approved by the ethics committee of our hospital, written informed consent was obtained from all patients prior to study participation. This study was registered in Chinese Clinical Trial Registry with the number ChiCTR-OOC-16009450. Patients were selected if they met the following criteria: clinical diagnosis of lung cancer limited to one lung, age 18–75 years, body mass index (BMI) 18–30 kg/m^2^, and American Society of Anesthesiologists physical status grade (ASA) I-II. The exclusion criteria were severe cardiovascular disease; second- or third-degree atrioventricular block on electrocardiography; history of renal, endocrine or neurological diseases; preoperative oral analgesic drugs; alcoholism; and pregnancy.

We enrolled 94 patients who were randomly divided into either a dexmedetomidine group or a normal saline group. In the dexmedetomidine group, dexmedetomidine 0.8 μg/kg was administered for 10 min before anesthesia. The normal saline group received the same volume of normal saline.

### Anesthetic management

Patients received no premedication. After entering the operating room, patients were monitored using invasive BP (radial arterial BP), HR, pulse oximetry, and electrocardiography. The SPI value was measured using the Datex-Ohmeda S/5 ADU (GE Healthcare, Madison, WI) monitoring system. Peripheral venous access was established, and all patients underwent endobronchial anesthesia. Anesthesia induction involved intravenous fentanyl (4.0 μg/kg), propofol (2.0 mg/kg), and cisatracurium (0.15 mg/kg). Endobronchial intubation was performed once the injectable anesthetics were effective. One-lung ventilation was continued from skin incision to skin closure and using 100% oxygen. Intraoperative end-tidal carbon dioxide was maintained at 35–45 mmHg by adjusting the ventilation rate and tidal volume. Anesthesia was maintained with 2.0–3.0% isoflurane, fentanyl (2.0 μg/kg), and cisatracurium (0.10 mg/kg/h). During surgery, we ensured that patients´ BP and HR fluctuations did not exceed 20% of their preoperative baseline values by modulating the narcotic drugs that we administered. Intraoperative anesthetic drug administration still depended on conventional empirical approaches according to changes in BP and HR to maintain anesthesia depth. If HR fell below 50 bpm, we administered 0.01 mg/kg atropine intravenously. If systolic pressure dropped below 90 mmHg, we administered 0.1 mg/kg ephedrine intravenously. Following surgery, all patients were transported to the post anesthesia care unit (PACU).

### Data collection

SPI, HR, and BP were recorded when the patient entered the operating room (baseline), at intubation, at the beginning of the operation (skin incision), end of the operation (skin closure), and at PACU discharge. The depth of sedation was assessed using the Observer’s Assessment of Alertness/Sedation (OAA/S) scale 10 min after extubation (5 = responds readily to name spoken in a normal tone; 4 = lethargic response to name spoken in a normal tone; 3 = responds only after name is called loudly or repeatedly; 2 = responds only after mild prodding or shaking; 1 = does not respond to mild prodding or shaking). Patients used the numerical rating scale (NRS) (0 = no pain and 10 = the worst pain) to rate their pain, and we evaluated the NRS scores at PACU discharge and 24 or 48 h after surgery.

### Statistical analysis

Data were analyzed using SPSS version 19.0 (IBM Corp., Armonk, NY). Continuous normally-distributed data were compared using Student’s t-test. Non-continuous and non-normally distributed data were analyzed using the Mann-Whitney test. Results were expressed as means ± standard deviations or as medians. Categorical variables were analyzed using the χ^2^ test, and *P* < 0.05 was considered statistically significant between groups.

## Results

### Patients’ characteristics

Four patients were excluded. One patient was excluded because of a significant decrease in HR (< 45 bpm) during surgery in the dexmedetomidine group, and three patients were excluded in the normal saline group. Two were excluded due to conversion to thoracotomy, and one was excluded due to major intra-operative bleeding. A final 90 patients were enrolled in the study; 46 in the dexmedetomidine group and 44 in the normal saline group. Patients’ baseline characteristics (age, sex, ASA status, weight, and BMI) did not differ between the two groups (Table 1). The length of surgery and anesthesia also did not differ between the two groups. Anesthetic dosages trended downward in the dexmedetomidine group, but there was no significant difference between the two groups. The total amount of propofol used, and the extubation time and Observer’s Assessment of Alertness/Sedation score in the PACU did not differ between the groups (Table 1).

**Table 1:** Patients’ baseline characteristics

	Dexmedetomidine group (*n* = 46)	Normal saline group (*n* = 44)	*P* value
Male/female (n)	17/29	22/22	0.212
ASA I/II (n)	7/39	10/34	0.391
Age (year)	56.78 ± 12.81	60.48 ± 12.58	0.171
Weight (kg)	58.48 ± 10.09	61.77 ± 8.71	0.101
BMI (kg/m^2^)	22.09 ± 3.22	22.89 ± 2.85	0.217
Duration of surgery (min)	162.37 ± 59.62	159.95 ± 60.59	0.849
Duration of anesthsia (min)	191.35 ± 62.26	203.27 ± 53.41	0.333
Fentanyl used during surgery (mg)	0.49 ± 0.07	0.52 ± 0.08	0.086
Propofol used during surgery (mg)	116.70 ± 20.30	123.75 ± 17.71	0.083
Time of extubation (min)	18.41 ± 11.97	17.59 ± 12.20	0.748
OAA/S	4.41 ± 0.54	4.59 ± 0.54	0.123

Data are presented as mean ± standard deviation or number

*ASA* American Society of Anesthesiologists, *BMI* body mass index, *OAA/S* Observer’s Assessment of Alertness/Sedation

### SPI value

**Fig. 1:**
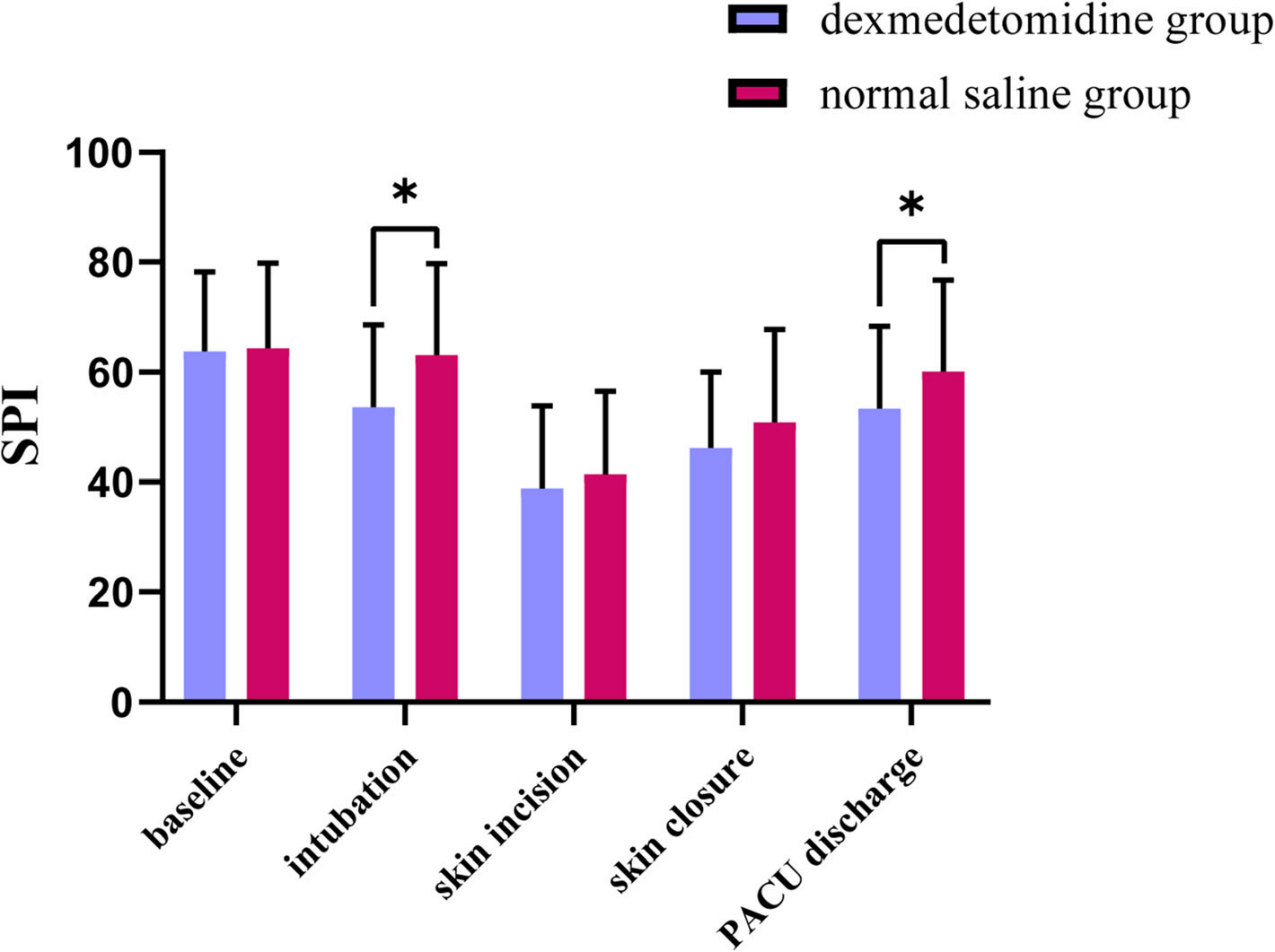
Comparison of SPI values between the two groups undergoing video-assisted thoracoscopic lung lobectomy from the time patients entered the operating room to when they left the recovery room. SPI: Surgical Pleth Index, PACU: post anesthesia care unit, *P < 0.05

SPI values showed no significant differences between the groups at baseline, at skin incision, and at skin closure. Compared with the normal saline group, SPI values in the dexmedetomidine group were lower at intubation (53.61 ± 15.03 vs. 63.14 ± 16.60, respectively; *P* = 0.005) and at PACU discharge (53.37 ± 14.98 vs. 60.11 ± 16.60, respectively; *P* = 0.046) (Fig. 1).

### Hemodynamic parameters

**Fig. 2:**
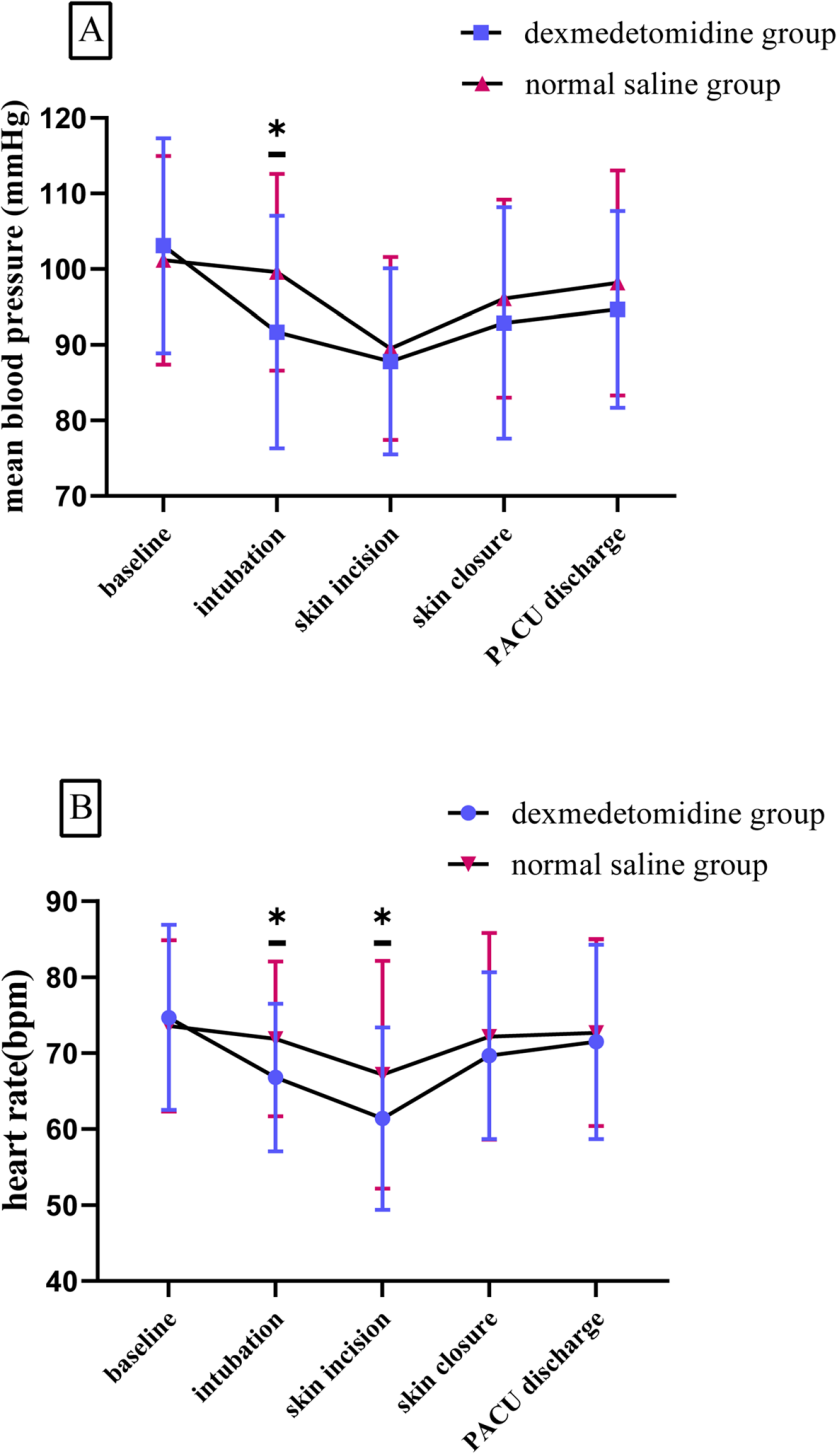
Hemodynamic changes in both groups. **a** Mean blood pressure at different time points, **b** Heart rate at different time points. PACU: post anesthesia care unit, *P < 0.05

Mean BP was higher in the normal saline group than in the dexmedetomidine group at intubation (99.57 ± 12.97 vs. 91.74 ± 15.37, respectively; *P* = 0.011) (Fig. 2). HR was lower in the dexmedetomidine group compared with the normal saline group at intubation (66.80 ± 9.66 vs. 71.86 ± 10.25, respectively; *P* = 0.018) and at skin incision (61.39 ± 12.05 vs. 67.20 ± 15.01, respectively; *P* = 0.045) (Fig. 2).

### NRS pain score

**Fig. 3:**
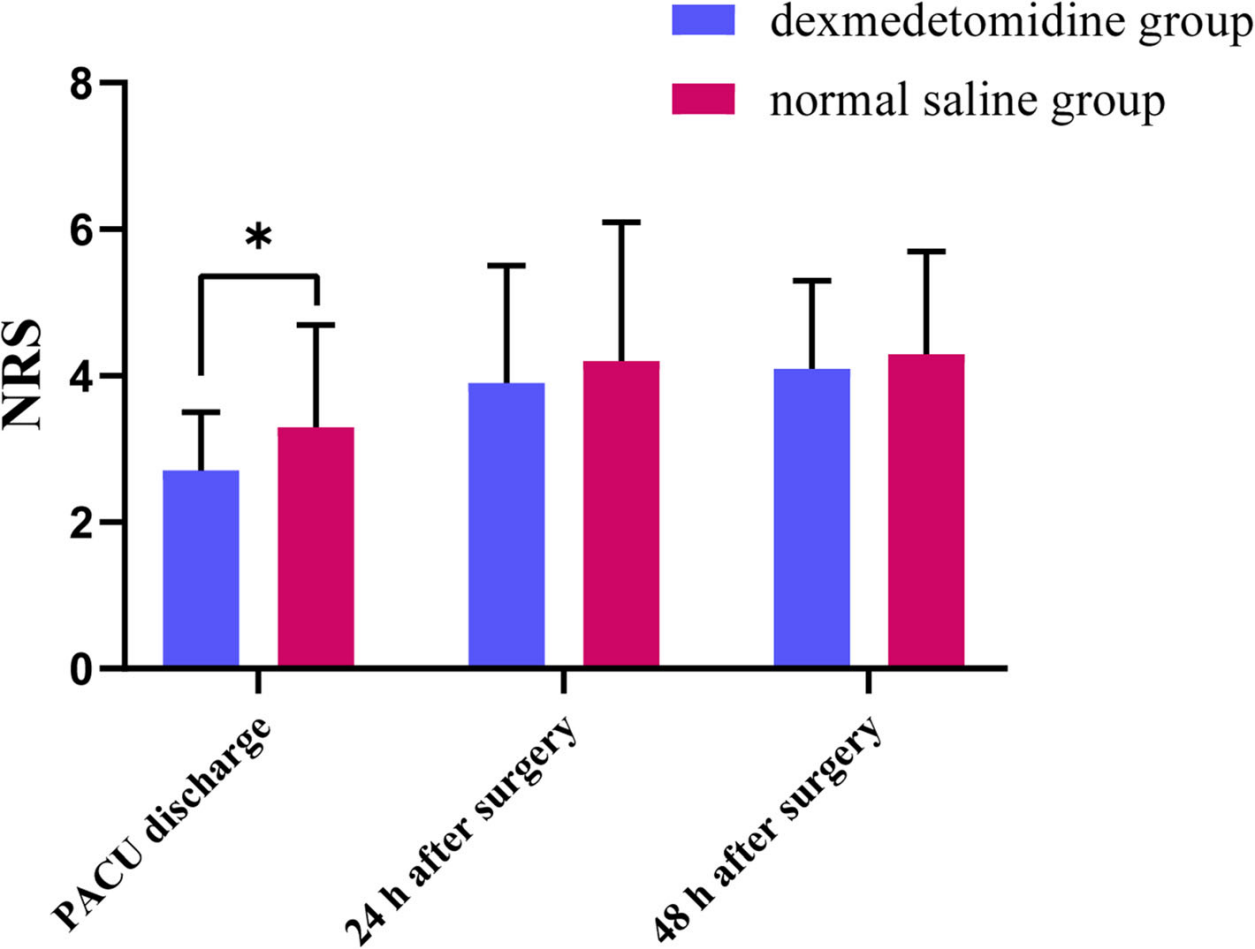
Comparison of postoperative NRS pain scores between the two groups from when they left the recovery room to 48 h after surgery. NRS: number rating scale, PACU: post anesthesia care unit, *P < 0.05

Surgical resections were performed using a two-port approach in the two groups, and a chest tube (28 Fr) was placed at the end of the surgery. The NRS pain scores in the normal saline group were higher than in the dexmedetomidine group at PACU discharge (3.25 ± 1.37 vs. 2.67 ± 0.82, respectively; *P* = 0.018). However, there were no remarkable differences in pain scores between the two groups 24 or 48 h postoperatively (Fig. 3).

## Discussion

In this study, dexmedetomidine administered before anesthesia decreased patients’ SPI values and pain scores, and dexmedetomidine led to more stable intraoperative hemodynamics.

VATS has been widely used to treat lung cancer because it is minimally invasive, more effectively decreases postoperative pain compared with open thoracotomy, and shortens hospital stay [12]. However, postoperative pain management, particularly early postoperative pain, remains a matter of concern for many anesthesiologists [2, 13]. Opioids are essential during surgery; however, reducing opioid consumption has become important because of their side effects, such as delayed recovery from general anesthesia, opioid-induced nausea, and respiratory depression [14, 15]. Reducing the use of opioids during surgery improves patients’ postoperative recovery. Intraoperative dexmedetomidine improves the effects of postoperative analgesia [16–18], and as an adjunct to general anesthesia, dexmedetomidine has analgesic, sedative, and anxiolytic effects, and avoids respiratory depression and the inhibitory effect of sympathetic stimulation [8, 9]. Dexmedetomidine maintains the stability of the cardiovascular system and decreases the stress response [10]. Many studies have shown that dexmedetomidine has opioid-sparing properties [19, 20] and that it maximizes pain relief and minimizes analgesic-related side effects [21]. In this study, dexmedetomidine used before anesthesia decreased NRS scores in the PACU. Although dexmedetomidine did not decrease the amount of fentanyl required, we saw a trend towards less fentanyl use.

SPI is calculated by heart beat intervals (HBI) and PPGA, both of which are obtained from pulse oximetry [4]. SPI values, which indicate surgical stress reactions, range between 0 and 100, with 0 representing the lowest stimulus response and 100 representing the highest level of stimulation. High SPI values are considered indicators of a prevalence of nociception over antinociception [7]. Surgical procedures under general anesthesia elicit a variety of stress responses that induce negative influences and that eventually negatively affect patients’ prognosis [22, 23]. SPI reflects the relationship between surgical stimulation intensity and antinociception during general anesthesia; higher values indicate stronger stimuli [4, 22, 24–26]. Therefore, SPI is more predictable for measuring autonomic responses to noxious stimuli and providing effective analgesia [22, 23]. Surgical stimulation affects the sympathetic nerves, causing changes in plethysmographic pulse waves and HR, which lead to changes in SPI. Because dexmedetomidine decreases the stress response, it also decreases HR, and lower HRs increase HBI values according to the following formula: SPI = 100 − (0.33 × HBInorm + 0.67 × PPGAnorm) [4]. Dexmedetomidine decreased HR at intubation, in this study, resulting in significantly lower SPI at the same time point. Dexmedetomidine inhibits the stress of tracheal intubation, and stabilizes circulation; however, even though HR was notably lower at the beginning of surgery with dexmedetomidine, SPI values did not differ between the two groups. This finding might indicate that stress stimulus intensity was stronger than HR reduction at skin incision. Ahonen et al. [27] reported that beta blockers decrease HR, only, and do not blunt the nociceptive response. If nociceptive stimuli exceed the effect of lower HR on SPI, then SPI does not necessarily decrease, which explains why the SPI value was not affected at the beginning of surgery, in our study. The pharmacological properties of dexmedetomidine include an auxiliary analgesic effect and stress response suppression, and dexmedetomidine significantly decreased the SPI value in the PACU, in our study. Therefore, postoperative adverse stimuli were well suppressed.

Previous studies indicated that SPI is correlated with NRS [3, 26, 28]. In the study, dexmedetomidine decreased SPI values. Dexmedetomidine decreased the surgical stress response and also relieved postoperative pain secondary to its analgesic effect. Therefore, SPI and NRS decreased simultaneously in the PACU. The reduction in patients’ pain indicated that the surgical stress response was well suppressed, and SPI values were lower, indicating a correlation between SPI and NRS.

There are two main limitations in this study. First, dexmedetomidine was used only with a loading dose before anesthesia. Results may change with continuous dexmedetomidine infusion during surgery following the loading dose. Second, we did not measure stress indicators such as catecholamines during surgery. Our future research plans involve addressing these limitations.

## Conclusion

Dexmedetomidine decreased surgical stress and postoperative pain during moderate-intensity surgery in VATS lung lobectomy. Our results demonstrated that dexmedetomidine during general anesthesia attenuated noxious stimulation.

## Data Availability

The datasets used and analysed during the current study are available from the corresponding author on reasonable request.

## Abbreviations

SPI: Surgical pleth index
NRS: Number rating scale
VATS: Video-assisted thoracoscopic surgery
PPGA: Photoplethysmographic amplitude
HR: Heart rate
BMI: Body mass index
ASA: American Society of Anesthesiologists physical status grade
BP: Blood pressure
PACU: Post anesthesia care unit
OAA/S: Observer’s Assessment of Alertness/Sedation
HBI: Heart beat intervals
